# Harmonizing organ-at-risk structure names using open-source large language models^☆^

**DOI:** 10.1016/j.phro.2025.100813

**Published:** 2025-07-24

**Authors:** Adrian Thummerer, Matteo Maspero, Erik van der Bijl, Stefanie Corradini, Claus Belka, Guillaume Landry, Christopher Kurz

**Affiliations:** a Department of Radiation Oncology, LMU University Hospital, LMU Munich, Munich, Germany; b Department of Radiation Oncology, University Medical Center Utrecht, Utrecht, the Netherlands; c Department of Radiation Oncology, Radboud University Medical Center, Nijmegen, the Netherlands; d German Cancer Consortium (DKTK), partner site Munich, a partnership between DKFZ and LMU University Hospital Munich Germany, Munich, Germany; e Bavarian Cancer Research Center (BZKF), Munich, Germany

**Keywords:** Large language models, LLMs, Structure renaming, AAPM TG-263

## Abstract

**Background and purpose:**

Standardized radiotherapy structure nomenclature is crucial for automation, inter-institutional collaborations, and large-scale deep learning studies in radiation oncology. Despite the availability of nomenclature guidelines (AAPM-TG-263), their implementation is lacking and still faces challenges. This study evaluated open-source large language models (LLMs) for automated organ-at-risk (OAR) renaming on a multi-institutional and multilingual dataset.

**Materials and methods:**

Four open-source LLMs (Llama 3.3, Llama 3.3 R1, DeepSeek V3, DeepSeek R1) were evaluated using a dataset of 34,177 OAR structures from 1684 patients collected at three university medical centers with manual TG-263 ground-truth labels. LLM renaming was performed using a few-shot prompting technique, including detailed instructions and generic examples. Performance was assessed by calculating renaming accuracy on the entire dataset and a unique dataset (duplicates removed). In addition, we performed a failure analysis, prompt-based confidence correlation, and Monte Carlo sampling-based uncertainty estimation.

**Results:**

High renaming accuracy was achieved, with the reasoning-enhanced DeepSeek R1 model performing best (98.6 % unique accuracy, 99.9 % overall accuracy). Overall, reasoning models outperformed their non-reasoning counterparts. Monte Carlo sampling showed a stronger correlation with prediction errors (correlation coefficient of 0.70 for DeepSeek R1) and better error detection (Sensitivity 0.73, Specificity 1.0 for DeepSeek R1) compared to prompt-based confidence estimation (correlation coefficient < 0.42).

**Conclusions:**

Open-source LLMs, particularly those with reasoning capabilities, can accurately harmonize OAR nomenclature according to TG-263 across diverse multilingual and multi-institutional datasets. They can also facilitate TG-263 nomenclature adoption and the creation of large, standardized datasets for research and AI development.

## Highlights

• Large language models can rename structures according to the AAPM TG-263 guideline.
• Investigated four open source models using a multi-lingual, multi-center dataset.
• DeepSeek R1 achieved the highest accuracy with 98.6% correctly renamed structures.
• Monte Carlo uncertainty correlated with prediction errors (r = 0.7)

## 1 Introduction

In radiation oncology, accurate delineation of target volumes and organs-at-risk (OARs) is critical for effective treatment planning, balancing precise dose delivery to targets while sparing healthy tissues. However, significant variability in naming conventions for radiotherapy structures persists within and between institutions and countries [1]. Recognizing the need for standardization of radiotherapy structure names, in 2018 the American Association of Physicists in Medicine (AAPM) established Task Group 263 (TG-263) to provide comprehensive guidelines for harmonizing nomenclature [2]. Despite this effort, adoption remains limited due to legacy datasets, institutional and vendor variability, perceived limited benefit, and resource constraints [3]. Overcoming these barriers is critical to fully leverage the advantages of standardized datasets, particularly for developing advanced artificial intelligence applications that rely on large, consistently labeled datasets.

Several approaches have been proposed to automate structure renaming according to the TG-263 standard, including machine learning (ML) pipelines utilizing anatomical data [4], natural language processing (NLP) techniques analyzing clinician-assigned names [5] and automated tools and scripts, some integrated within treatment planning systems, to check and enforce compliance with TG-263 nomenclature [6,7].

Recent advancements in NLP, particularly in large language models (LLMs), present new possibilities for automating complex language-related tasks within the medical domain [[8], [9], [10]]. LLMs trained on extensive textual datasets have demonstrated remarkable capabilities in understanding and generating human-like text, making them also relevant for applications in radiation oncology [[11], [12], [13]]. A recent study by Holmes et al. has demonstrated the potential of LLMs for renaming radiotherapy structures in alignment with the TG-263 guideline [14], utilizing openAI’s proprietary GPT4 model for renaming OAR and target structures in prostate, head-and-neck and thoracic cancer patients, achieving high renaming accuracies up to 98.3 % [15], but limited to a single-institution dataset in English and center-specific prompts.

Our study evaluated open-source LLMs for radiotherapy structure renaming using a multilingual, multi-institutional dataset, comprising 34,177 radiotherapy structures from three European university medical centers. We focused specifically on open-source LLMs which can be deployed locally, thereby addressing critical privacy considerations within healthcare environments. Performance evaluation included comparison against manually assigned ground-truth labels, detailed error analysis, and uncertainty estimation, ensuring robust and reliable automated renaming workflows.

## 2 Materials and methods

**Fig. 1:**
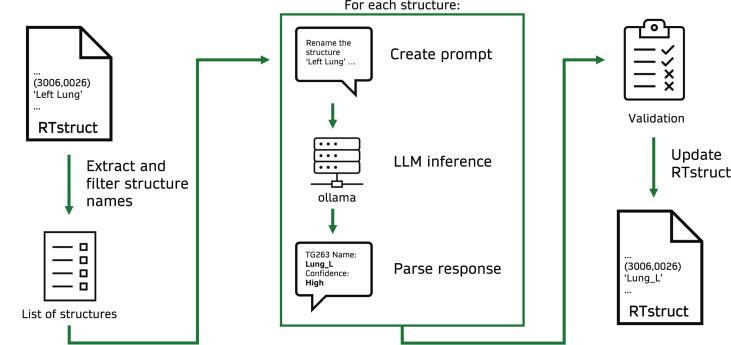
Schematic overview of the envisioned renaming pipeline.

The proposed LLM-based renaming workflow is illustrated in Fig. 1. Original structure names were extracted from DICOM RTstruct files and filtered to exclude non-TG-263 structures (see 2.1). For each structure, a prompt containing renaming instructions (see 2.2) was generated and used as input for the LLMs. Each LLM predicted a TG-263-conform structure name and an associated confidence score (see 2.6). All pipeline components were implemented in Python, including a graphical user interface to load, filter and review renamed structures (see Fig. S1 in supplementary materials). The source code is available at https://github.com/LMUK-RADONC-PHYS-RES/rt-rename.

### 2.1 Dataset

We utilized a fully anonymized dataset curated for the SynthRAD2025 deep learning challenge [16], containing structure information from 1,684 radiotherapy patients treated for head-and-neck, thoracic or abdominal cancer at three European medical centers: University Medical Center Utrecht, Radboud University Medical Center Nijmegen (both in the Netherlands), and LMU Klinikum Munich (Germany). These institutions did not adopt the TG-263 nomenclature, resulting in center-specific structure names in English, Dutch, or German. Our analysis focused explicitly on organs-at-risk (OAR). We manually excluded target structures, auxiliary planning structures (e.g., couch, immobilization masks, etc.) and OARs not part of the TG-263 guideline. Ground-truth labels were assigned manually for each structure by staff from the respective institutions, yielding a total dataset of 34,177 structures. Additionally, a refined list of 1,094 structures was generated by removing duplicates, facilitating more efficient inference, since duplicate structures were not renamed repeatedly. Evaluations were conducted on both the complete and unique datasets.

### 2.2 Prompt

The input prompt was iteratively developed using a small dataset comprising one patient from each center and included: (1) general instructions outlining the renaming task; (2) rules about possible languages (English, German, Dutch), abbreviations, anatomical locality indicators (e.g left, right, superior, inferior) and instructions to reply with *“no match”* if no suitable structure name was found; (3) instructions for output formatting and confidence level output (low, medium or high); (4) a few-shot prompting strategy with examples of both input and expected output, as this approach has been shown to enhance LLMs’ task comprehension and adherence to format [17,18]; (5) a list of all standardized TG-263 structure names [2] and the input structure name to be processed. Each prompt contained only a single input structure name per renaming task. The final prompt is provided in supplementary materials (Fig. S2).

### 2.3 Models

This study evaluated four open-source LLMs: Llama 3.3, Llama 3.3 R1, DeepSeek V3, and DeepSeek R1.

Llama 3.3 70B is a pre-trained and instruction-tuned LLM from Meta AI, comprising 70 billion parameters [19]. This multilingual model officially supports eight languages, including English and German (but not Dutch) and features a context window of up to 128 k tokens. Tokens are a unit of text, such as a word, sub-word or character, that the LLM processes as a single input element during training and inference [20]. Due to GPU memory limitations (NVIDIA A6000, 48 GB), a 4-bit quantized model (Q4_K_M) was used for inference. A deterministic output was ensured by setting the temperature parameter to 0 for all LLMs.

DeepSeek V3 is a pre-trained, instruction-tuned Mixture-of-Experts (MoE) LLM developed by DeepSeek-AI [21]. It comprises 671 billion parameters, with 37 billion activated per token, making it significantly larger than the Llama 3.3 70B model. Due to its size, inference of the V3 model was performed on a cloud platform with unspecified hardware using an unquantized model. Deepseek V3 also features a 128 k token context window.

DeepSeek R1 is a reasoning-specialized LLM from Deepseek AI, explicitly trained for logical reasoning using the chain-of-thought (CoT) methodology. It employs the same MoE architecture as DeepSeek V3, with 671 billion total parameters and 37 billion active per token, and a 128 k token context window [22]. The CoT approach generates more tokens during inference, increasing inference time and computational cost. Inference was performed on the same cloud platform as for DeepSeek V3.

Llama 3.3 70B R1 is a distilled variant of the original Llama 3.3 70B model, fine-tuned on outputs from the larger DeepSeek R1 671B model [22]. This distillation enhances the reasoning capabilities of the smaller Llama 3.3 70B model, especially in complex reasoning tasks involving CoT [22]. Like its base model, Llama 3.3 70B R1 supports the same context window of 128 k tokens, and we used a 4-bit quantized model (Q4_K_M) on a NVIDIA A6000 GPU.

### 2.4 Accuracy

Renaming accuracy was evaluated by comparing LLM predictions to manually assigned TG-263 labels. The evaluation was structured as a classification task, with each structure instance assigned a single correct label. Two accuracy metrics were calculated: (1) unique match accuracy, the proportion of exact matches between LLM-predicted and ground-truth labels across 1094 distinct structure names; and (2) overall accuracy, calculated on the full dataset of 34,177 structure names including all instances and duplicates. Accuracy was reported per center and for the combined dataset.

### 2.5 Failure analysis

Misassignments were manually classified into five categories: (1) *Wrong OAR* (incorrect organ-at-risk identified); (2) *Laterality* (correct OAR, incorrect spatial designation, e.g., left vs. right, superior vs. inferior); (3) *Plurality* (correct OAR, singular/plural form errors); (4) *Misspelling* (correct OAR, misspelling of TG-263 name); and (5) *No match* (no suitable TG-263 name found). Error frequencies were computed per LLM for the unique dataset, with representative examples provided in the results section.

### 2.6 Prompt-based confidence estimation

The input prompt contained instructions for a confidence rating (low, medium, or high) next to the predicted structure names. To assess the usability of these self-reported confidence ratings as indicators of naming errors, we calculated a binary Pearson correlation coefficient, between low confidence ratings (low or medium = 1, high = 0) and structure naming errors (false = 1, correct = 0) for the unique dataset. This quantified the usefulness of self-reported confidence for flagging errors.

### 2.7 Monte Carlo sampling for uncertainty estimation

A temperature value of 0 enforces deterministic behavior by consistently selecting the most probable token at each step, yielding identical outputs for repeated inferences. In contrast, higher temperature values introduce stochastic behavior by allowing less probable tokens to be selected, enabling more diverse responses. We used a Monte Carlo sampling approach to assess the uncertainty associated with structure name predictions. Monte Carlo sampling and uncertainty estimation is a framework for quantifying uncertainty through repeated randomized simulations and has been widely applied in radiotherapy for tasks such as image synthesis or auto-segmentation [[23], [24], [25]]. We performed ten repeated inferences for each input structure using a temperature of 1 in combination with top-p sampling (0.95). This sampling strategy samples from the smallest set of tokens whose cumulative probability exceeds 95 %. Due to computational demands of repeated inferences, we limited the analysis to a randomly selected subset of 30 structures per model, comprising 15 correctly and 15 incorrectly labeled cases under deterministic conditions.

For each structure, we calculated the Shannon entropy of the distribution of predicted labels, defined as:

$$H\left(p\right)=-\sum_{i=1}^{n}p_{i}\log p_{i}$$

where $p_{i}$ denotes the relative frequency of the i-th unique predicted structure across the n distinct predicted structures. Entropy is a quantitative measure of uncertainty and output variability in this context. We calculated the binary Pearson correlation coefficient to quantify the correlation between wrong structure name assignments (wrong = 1, correct = 0) and non-zero entropy. Additionally, we calculated the sensitivity (true positive rate) and specificity (true negative rate) for using entropy as a binary classifier of prediction errors, where a positive event was defined as an incorrect structure name assignment with corresponding non-zero entropy.

## 3 Results

### 3.1 Accuracy

**Fig. 2:**
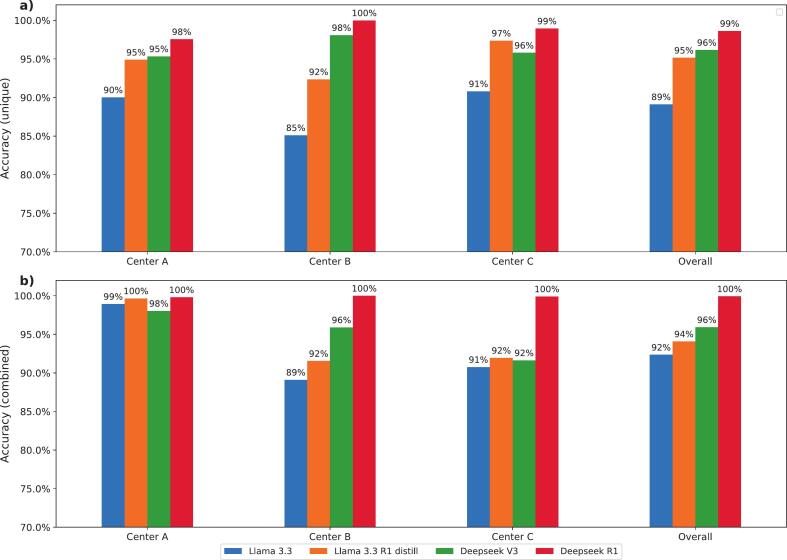
Renaming accuracy of Llama 3.3, Llama 3.3 R1 distill, Deepseek V3 and Deepseek R1 for datasets from center A, B and C calculated for the unique (a, upper) and combined datasets (b, lower).

Deepseek R1, which incorporates reasoning capabilities, consistently achieved the highest accuracy on the unique dataset (Fig. 2a), with center-specific accuracies ranging from 97.6 % to 100 %. In contrast, the Llama 3.3 model, lacking reasoning functionality, showed the lowest accuracy with values ranging from 85.1 % to 90.8 %. The results revealed variability across centers on the complete dataset (Fig. 2b). For Center A, all four models showed improved accuracy relative to their performance on the unique dataset, ranging from 98.0 % (DeepSeek V3) to 99.9 % (DeepSeek R1). Contrarily, for Center B, accuracy declined for DeepSeek V3 and Llama 3.3 R1, while Llama 3.3 improved and DeepSeek R1 remained constant with a perfect 100 % accuracy. A declined accuracy indicates that failed structures had a high frequency in the overall dataset. Results from Center C also showed mixed outcomes, with Llama 3.3 and DeepSeek R1 slightly improving the accuracy, while Llama 3.3 R1 and DeepSeek V3 experienced slight reductions in accuracy.

Tables S1 and S2 in the supplementary materials present the corresponding fractions of correctly renamed structures. To further explore model performance across anatomical regions, we conducted a sub-analysis grouped by anatomical region: head-and-neck, thorax, and abdomen. Results are presented in Fig. S3 in the supplementary materials. All four LLMs achieved their highest accuracy on abdominal structures and their lowest on thoracic structures. Fig. S4 in the supplementary materials presents some exemplary outputs from the Llama 3.3 R1 and DeepSeek R1 reasoning models, illustrating the CoT reasoning process.

Inference times for locally deployed models averaged 1.2 s for Llama 3.3 and 25.1 s for Llama 3.3 R1 using a single NVIDIA A6000 GPU with 48 GB VRAM. Average inference times on the cloud-hosted inference platform were 1.5 s for DeepSeek V3 and 35.6 s for DeepSeek R1.

### 3.2 Error types

**Fig. 3:**
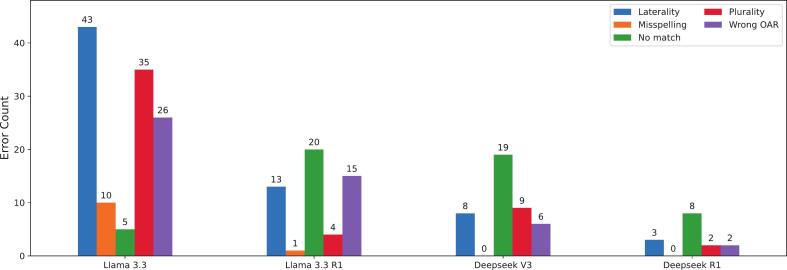
Distribution of error types for each LLM based on misclassified structure names.

Table 1 presents representative examples of each error type. For the Llama 3.3 model, laterality, plurality, and wrong OAR errors were recurring most often, while “no match” errors were rare, indicating an overly confident model (Fig. 3). In contrast, the “no match” error was the most frequently observed error type for the Llama 3.3 R1, Deepseek V3, and Deepseek R1 models. Misspelling errors were also mainly observed in the Llama 3.3 model.

**Table 1:** Examples of the error types observed in the renamed structure names.

Error type	Model	Input structure	LLM prediction	GT	Comments
Locality	Llama 3.3	*CervMyelum*	*SpinalCord*	*SpinalCord_Cerv*	Correctly identified “SpinalCord” but failed to capture the regional specification “Cerv”.
Plurality	Llama 3.3 R1	*Niere bds*	*Kidney*	*Kidneys*	Correctly translated “Niere” (kidney in German), but failed to interpret “bds” (bilateral).
Wrong OAR	Deepseek V3	*PharynxConst*	*Pharynx*	*Musc_Constrict*	Misinterpreted “PharynxConst” as general pharynx rather than pharyngeal constrictor muscles.
No match	Deepseek R1	*Sfincter*	*No match*	*Sphincter_Anal*	Failed to identify and match “Sfincter” with the correct anatomical term.
Misspelling	Llama 3.3	*Traenendruese li.*	*Lacrimal_Gland_L*	*Glnd_Lacrimal_L*	Correctly identified the OAR but did not adhere to the required TG-263 spelling.

### 3.3 Prompt-based uncertainty estimation

**Fig. 4:**
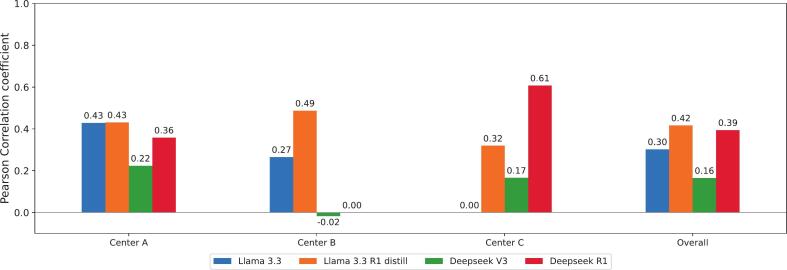
Pearson correlation coefficient calculated between low confidence (low or medium) and incorrect structure assignment for the four investigated LLMs grouped by centers.

Only a moderate positive correlation was observed between confidence levels (low or medium) and prediction errors. Fig. 4 presents the corresponding Pearson correlation coefficients. For the combined dataset, the reasoning models Llama 3.3 R1 and DeepSeek R1 exhibited the strongest correlation between reduced confidence and incorrect predictions, with correlation coefficients of 0.42 and 0.39, respectively. The non-reasoning Llama 3.3 and Deepseek V3 models showed noticeably lower correlation coefficients of 0.30 and 0.16 respectively.

### 3.4 Monte Carlo sampling for uncertainty estimation

Deepseek R1 achieved the highest correlation coefficient (0.70) between non-zero entropy and structure renaming failures, suggesting that entropy can be a useful metric to flag potentially wrong structure name assignments (Table 2). This model also demonstrated the highest sensitivity (0.73) and perfect specificity (1.0). Similar to the analysis of prompt-based confidence levels, reasoning models (Deepseek R1 and Llama 3.3 R1) showed a higher correlation between entropy and errors than non-reasoning models (Deepseek V3 and Llama 3.3).

**Table 2:** Performance metrics reported as Pearson correlation coefficient, sensitivity (true positive rate = TPR) and specificity (true negative rate = TNR) for entropy-based uncertainty estimation derived from Monte Carlo sampling.

Model	Correlation coefficient	Sensitivity (TPR)	Specificity (TNR)
Llama 3.3	0.18	0.07	1
Llama 3.3 R1	0.57	0.67	0.87
Deepseek V3	0.36	0.64	0.36
Deepseek R1	0.70	0.73	1

## 4 Discussion

This study systematically evaluated open-source large language models (LLMs) for harmonizing radiotherapy OAR structure nomenclature according to the AAPM TG-263 guideline across a multilingual, multi-institutional dataset. To our knowledge, this is the first comprehensive assessment of open-source LLMs in this context, addressing generalizability across institutions and languages and data privacy concerns. Our results demonstrated that open-source LLMs can effectively standardize structure names, with DeepSeek R1 achieving the highest accuracy (99 %).

Performance was comparable to Holmes et al., who used a proprietary model with center-specific prompts and a single center dataset and achieved an accuracy of 97 % (including target volumes) [14]. The strong performance of DeepSeek R1 and the distilled Llama 3.3 R1 highlights the benefit of explicit reasoning capabilities, particularly in ambiguous or abbreviated structure names. Despite identical architecture, Llama 3.3 R1 outperformed the non-reasoning Llama 3.3 noticeably (95 % vs. 89 %). However, with a considerably longer inference time (1.2 s vs. 25.1 s), which could be further optimized by using a different LLM engine, batched inference, or higher-tier GPUs.

Analysis of error types revealed noticeable performance differences between the models, primarily linked to their size and reasoning capabilities. The smallest model (Llama 3.3) struggled mostly with “post-processing” tasks like assigning location identifiers, laterality, plurality, or interpreting abbreviations. In contrast, larger (DeepSeek V3) and reasoning enabled models (DeepSeek R1 and Llama 3.3 R1) were more robust, frequently opting for “no match” in ambiguous cases. This conservative approach is clinically advantageous, as false positives pose greater risk than unassigned labels flagged for review. Holmes et al. similarly noted that most errors in their study arose from minor spelling and capitalization errors [14]. However no detailed error analysis was performed in their study. We observed no clear correlation between structure name language and error type. In reasoning-capable LLMs, the CoT outputs could be particularly helpful for understanding why the model fails on an individual structure level.

Unlike previous studies [4,6,7,14], we also explored strategies for error prediction and quality assurance. Prompt-based confidence scores showed limited effectiveness in identifying uncertain cases, indicating that they are unsuitable for flagging uncertain cases for manual review in automated workflows. However, we observed that both reasoning models showed a higher correlation than the non-reasoning models, suggesting that the additional tokens generated in reasoning models can assist with confidence prediction. Monte Carlo sampling entropy demonstrated higher predictive correlation but significantly increased inference times, especially limiting real-time clinical application. For offline data processing, the observed inference times (1–35 s) remain acceptable, with expected hardware advancements further reducing limitations.

The proposed automatic renaming workflow directly addresses one of the primary roadblocks to TG-263 adoption: the perceived effort required to update institutional templates and legacy datasets [3]. By providing a reliable method for harmonizing existing structure sets and templates, our tool can substantially lower the barrier for clinical sites to implement standardized nomenclature and thereby facilitate the creation of large, consistently labeled datasets accelerating multi-institutional research and the development of advanced AI applications. The pipeline is also highly flexible and can be easily adapted by incorporating additional LLMs, custom prompts, or updated guidelines, such as those currently in preparation for TG-263.

However, standardizing nomenclature alone does not resolve the broader challenge of collecting and curating large multi-institutional datasets. Our work complements frameworks like that described by Refsgaard et al. for collecting and curating large scale datasets (e.g. a national dataset of over 7000 breast cancer patients) [26].

Previous efforts to automate TG-263 nomenclature standardization have evolved from machine learning methodologies that rely solely on image-based features, to multi-view approaches that integrate both image and text data for improved performance [4,5]. Our study demonstrates that due to rapid advancements in natural language processing, modern LLMs are powerful enough to make accurate, text-only structure renaming feasible, even on a complex multi-lingual and multi-institutional dataset. However, to address the challenging cases where text-based context alone may be insufficient, future work could explore advanced vision-language models (VLMs) [27].

There are several limitations in our study. Although our dataset was large and institutionally diverse, it included only three centers from two European countries. Future studies should investigate generalizability across additional languages and naming conventions. Additionally, our study solely focused on OARs. As shown by the recent TG-263 survey [3], implementing the structure naming guideline for target volumes and planning structures (e.g., PTVs, PRVs) presents additional challenges due to their higher intra-institutional variability and center-specific clinical protocols. Lastly, although our prompt was carefully designed and few-shot examples were used, prompt engineering remains a critical component that can influence model performance, and further optimization specific to each model could lead to better outcomes for specific models. Due to limited availability of GPUs in our institution, only the smaller Llama 3.3 and Llama 3.3 R1 models could be deployed locally on our own hardware, while the larger Deepseek models were hosted on a cloud platform. However, given sufficient hardware, DeepSeek models could also be deployed locally.

In conclusion, this work presents a novel, open-source LLM-based framework for harmonizing radiotherapy structure nomenclature, achieving a high accuracy across a multilingual and multi-institutional dataset. This can help with the clinical implementation of structure naming guidelines and generate large-scale, high-quality datasets for research and development in radiation oncology.

## CRediT authorship contribution statement

**Adrian Thummerer:** Conceptualization, Methodology, Software, Formal analysis, Investigation, Data curation, Writing – original draft, Visualization. **Matteo Maspero:** Conceptualization, Methodology, Resources, Data curation, Writing – review & editing. **Erik van der Bijl:** Conceptualization, Methodology, Resources, Data curation, Writing – review & editing. **Stefanie Corradini:** Resources, Data curation, Writing – review & editing, Funding acquisition. **Claus Belka:** Resources, Data curation, Writing – review & editing, Funding acquisition. **Guillaume Landry:** Conceptualization, Methodology, Validation, Resources, Data curation, Writing – review & editing, Project administration, Funding acquisition. **Christopher Kurz:** Conceptualization, Methodology, Validation, Resources, Data curation, Writing – review & editing, Project administration, Funding acquisition.

## Declaration of competing interest

The authors declare that they have no known competing financial interests or personal relationships that could have appeared to influence the work reported in this paper. The author Guillaume Landry is an Editorial Board Member for this journal and was not involved in the editorial review or the decision to publish this article.
