# Supplementary material for: Harmonizing organ-at-risk structure names using open-source large language models
Source: Phys Imaging Radiat Oncol. 2025 Jul 24;35:100813. doi: 10.1016/j.phro.2025.100813 (PMC12336799; doi:10.1016/j.phro.2025.100813)
Supplement: Supplementary Data 1 [file mmc1.pdf]

Supplementary material

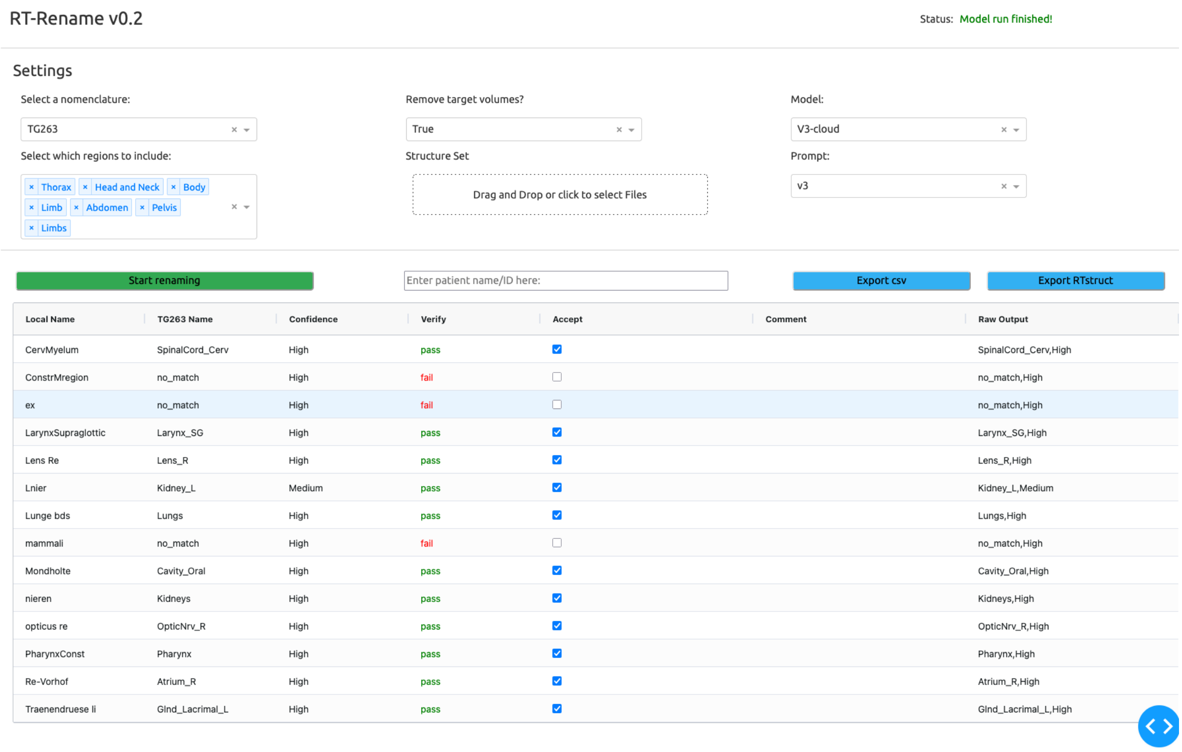

Local Name	TG263 Name	Confidence	Verify	Accept	Comment	Raw Output
CervMyelum	SpinalCord_Cerv	High	pass	☑		SpinalCord_Cerv,High
ConstrMregion	no_match	High	fail	☐		no_match,High
ex	no_match	High	fail	☐		no_match,High
LarynxSupraglottic	Larynx_SG	High	pass	☑		Larynx_SG,High
Lens Re	Lens_R	High	pass	☑		Lens_R,High
Lnier	Kidney_L	Medium	pass	☑		Kidney_L,Medium
Lunge bds	Lungs	High	pass	☑		Lungs,High
mammali	no_match	High	fail	☐		no_match,High
Mondholte	Cavity_Oral	High	pass	☑		Cavity_Oral,High
nieren	Kidneys	High	pass	☑		Kidneys,High
opticus re	OpticNrv_R	High	pass	☑		OpticNrv_R,High
PharynxConst	Pharynx	High	pass	☑		Pharynx,High
Re-Vorhof	Atrium_R	High	pass	☑		Atrium_R,High
Traenendruese li	Glnd_Lacrimal_L	High	pass	☑		Glnd_Lacrimal_L,High

Figure S1: Screenshot of the graphical user interface (GUI) used for renaming structure data, created in Python using plotly-dash.

You are an expert with radiation oncology knowledge and your task is to assign names to anatomical structures based on an international guideline, the TG263 list. The naming task is essential for standardizing radiation oncology practices across different institutions and to ensure safe patient treatments.

Here are some rules:
- I will provide you with the complete TG263 list and a structure to name.
- Reply only with the TG263 name and a confidence level for your answer (High, Medium, Low).
- Assign a confidence level of "High" if you are very certain about your answer, "Medium" if there are multiple matches and you chose one, and "Low" if it was very difficult to find a match.
- REPLY EXACTLY LIKE THIS: <predicted TG263_name>,<confidence_level>
- Some structures may not have a direct match in the TG263 list, in that case you can reply with: no_match,<confidence_level>
- The provided input structure name can be in English, German or Dutch.
- Pay close attention to additional information regarding the side (left, right, L, R, etc.) and specific regions (inferior, superior, medium, upper, lower, etc).
- Consider that the doctor may have used a German, Dutch or English name for the structure.
- The structure name may contain typos or abbreviations. Try to find the best match in the TG263 list.

Here are some naming examples:
- structure_input: R_Long --> your reply: Lung_R, High
- structure_input: Niere li --> your reply: Kidney_L, High
- structure_input: Darm --> your reply: Bowel, High
- structure_input: house --> your reply: no_match, High
- structure_input: evo_tree --> your reply: no_match, High
- structure_input: ConstrictMus_inf --> your reply: Musc_Constrict_I, High

Here is the TG263 structures list (in English):
{TG263_list}
The doctor named the structure (in English, German or Dutch):
{structure_input}
What standard name would you pick for this structure?

- It his highly important that you reply only in the format shown in the examples: predicted TG263_name,confidence_level.
- Do not add any text, explanation or summary.
- THIS IS HIGHLY IMPORTANT FOR SAVE [sic] PATIENT TREATMENTS!

Figure S2: The prompt used in this study.

	Center A	Center B	Center C	Combined
**Llama 3.3**	406/451 (90.0%)	223/262 (85.1%)	346/381 (90.8%)	975/1094 (89.1%)
**Llama 3.3 R1**	428/451 (94.9%)	242/262 (92.4%)	371/381 (97.4%)	1041/1094 (95.2%)
**Deepseek V3**	430/451 (95.3%)	257/262 (98.1%)	365/381 (95.8%)	1052/1094 (96.2%)
**Deepseek R1**	440/451 (97.6%)	262/262 (92.4%)	377/381 (99.0%)	1079/1094 (98.6%)

Table S1: Number of correctly renamed structures and the corresponding percentages for structure datsets from center A,B, and C using the unique dataset.

	Center A	Center B	Center C	Combined
**Llama 3.3**	10313/10423 (98.9%)	16671/18707 (89.1%)	4581/5047 (90.8%)	31565/34177 (92.4%)
**Llama 3.3 R1**	10388/10423 (99.7%)	17126/18707 (91.6%)	4641/5047 (92.0%)	32155/34177 (94.1%)
**Deepseek V3**	10219/10423 (98.0%)	17941/18707 (95.9%)	4625/5047 (91.6%)	32785/34177 (95.9%)
**Deepseek R1**	10403/10423 (99.9%)	18707/18707 (100%)	5043/5047 (99.9%)	34153/34177 (99.9%)

Table S2: Renaming accuracy of Llama 3.3, Llama 3.3 R1 distill, Deepseek V3, and Deepseek R1 for datasets from center A, B and C using the combined dataset.

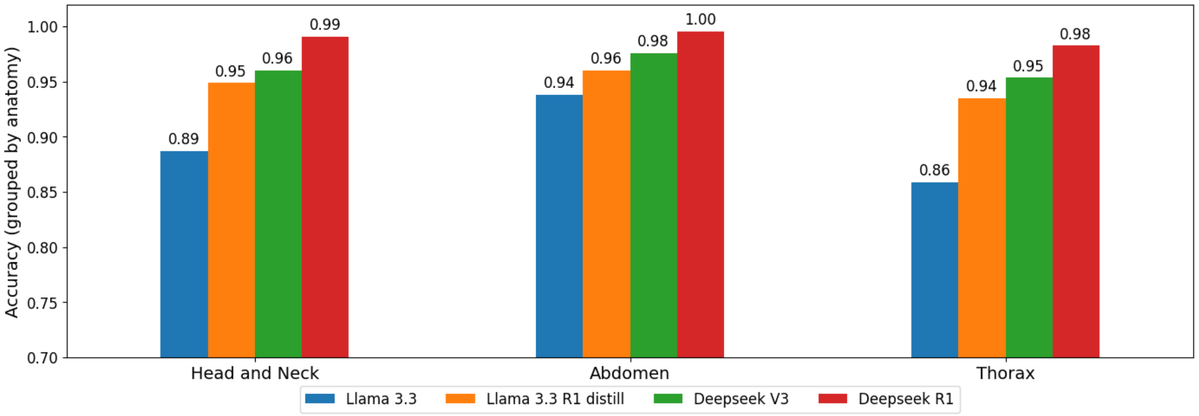

Figure S3: Renaming accuracy of Llama 3.3, Llama 3.3 R1 distill, Deepseek V3 and Deepseek R1 for datasets grouped by anatomical regions, calculated for the unique dataset (no duplicates).

Model: Llama 3.3 R1
Input structure name: Submandibularis_li
TG-263 conform name: Glnd_Submand_L

<think>
Okay, so I need to figure out the standard name for the structure "Submandibularis_li" using the TG263 list. Let me break this down step by step.
First, I recognize that "Submandibularis" refers to the submandibular gland. The "_li" part likely indicates the left side, as "li" is commonly used for "left" in medical abbreviations.
Looking through the TG263 list provided, I search for structures related to the submandibular gland. Scanning the names, I find entries like Glnd_Submand_L and Glnd_Submand_R, which stand for the left and right submandibular glands respectively.
Since "Submandibularis_li" specifies the left side, the correct match should be Glnd_Submand_L. This aligns with the structure's anatomical location and laterality.
I don't see any other structures in the list that would better fit this name, so I'm confident that Glnd_Submand_L is the right choice here.
</think>

Predicted TG263 name: Glnd_Submand_L
Confidence: High

Model: DeepSeek R1
Input structure name: Bijnier_re
TG-263 conform name: Glnd_Adrenal_R
<think>
Okay, let's tackle this. The input structure is "Bijnier_re". First, I need to figure out what this means. "Bijnier" is Dutch for "adrenal gland". The suffix "_re" might indicate the right side, since "re" could be short for "rechts" (right in Dutch). So translating directly, it's "Adrenal gland right".
Looking at the TG263 list, the adrenal glands are listed under "Glnd_Adrenal_L" and "Glnd_Adrenal_R". The naming convention here uses "Glnd" as an abbreviation for gland, followed by the organ name and side. Since the input specifies the right side, the correct TG263 name should be "Glnd_Adrenal_R".
I don't see any other possible matches in the list that would correspond to the adrenal gland. There's no alternative abbreviation or typo that fits better. The confidence here is high because the translation is straightforward and the TG263 entry matches exactly once the language is accounted for.
</think>

Predicted TG263 name: Glnd_Adrenal_R
Confidence: High

Figure S4: Exemplary chain-of-thought output from the Llama 3.3 R1 and DeepSeek R1 reasoning models.
